# Effects of TRX suspension training versus traditional balance training on balance performance in elite surfers

**DOI:** 10.1186/s13102-025-01411-z

**Published:** 2025-11-24

**Authors:** Zhaoyi Wang, Yong Ma, Qian Huang, Zhihao Guo, Mengyao Jia, Weitao Zheng

**Affiliations:** 1 School of Intelligent Sports Engineering, Wuhan Sports University, No. 461 Luoyu Road, Wuhan, Hubei Province 430079 China; 2 Key Laboratory of Sports Engineering of General Administration of Sport of China, Wuhan Sports University, Wuhan, Hubei China; 3 Specialised Research Centre for High-Quality Development of Competitive Sports, Wuhan Sports University, Wuhan, Hubei China; 4 Engineering Research Center of Sports Health Intelligent Equipment of Hubei Province, Wuhan Sports University, Wuhan, Hubei China

**Keywords:** Suspension training, Surfing, Static balance, Dynamic balance, Indo board lateral squat

## Abstract

**Objective:**

Traditional balance training (TB) may have certain limitations in replicating surfing’s unstable conditions, which may somewhat restrict its sport-specific efficacy. Total Resistance Exercise (TRX) suspension training uses controllable instability to better enhance key balance abilities. This study compared TRX and TB effects on static, dynamic, and surfing-specific balance in elite surfers, and explored correlations between these dimensions to inform targeted training.

**Methods:**

32 surfers from the current Chinese National Surfing Team were randomized to TRX or TB groups (*n* = 16 each). Both groups completed 30-minute training sessions ×3/week for 8 weeks. Static balance (eyes-closed single-leg stand), dynamic balance (Star Excursion Balance Test (SEBT), Linear travel deviation test), and surfing-specific balance (Indo board lateral squat) were assessed pre- and post-intervention. Pearson correlations examined relationships between balance dimensions.

**Results:**

(1) Both methods improved static balance (*p* < 0.01), but TRX group exhibited greater improvements (*p* < 0.001). (2) Dynamic balance improved significantly in both groups (*p* < 0.01), but TRX outperformed TB (SEBT: *p* = 0.034 for left leg, *p* = 0.043 for right leg; Linear travel deviation: *p* = 0.000). (3) Surfing-specific balance improved significantly in both groups (*p* < 0.01), with TRX superior to TB (*p* < 0.01); (4) Surfing-specific balance changes correlated significantly with static balance (moderate positive: *r* = + 0.487 for left leg, r = + 0.457 for right leg), multidirectional dynamic balance (weak positive: r = + 0.395 for left leg, r = + 0.363 for right leg), and directional control (moderate negative: *r*=-0.515).

**Conclusion:**

TRX suspension training is more effective than TB in enhancing static, dynamic, and surfing-specific balance in elite surfers. Thus, TRX suspension training can serve as a preferred sport-specific training method for improving balance ability in elite surfers, providing practical support for them to cope with complex wave conditions in competitions and enhance competitive performance. TRX suspension training can be integrated into elite surfers’ programs, aiding injury prevention and competitive performance optimization. Correlations support a hierarchical model where static balance provides a foundation, dynamic balance enables adaptation, and directional control reflects surfing’s uniqueness—findings that inform scientific design of surfing-specific training.

**Trial registration:**

Retrospectively registered. Chinese Clinical Trial Registry (https//www.chictr.org.cn/). Registration number ChiCTR2500102873. State Successful. Reg Date 20,250,521 000000.

**Supplementary Information:**

The online version contains supplementary material available at 10.1186/s13102-025-01411-z.

## Introduction

Surfing is an internationally popular and physically demanding water sport that requires athletes to demonstrate exceptional dynamic balance control to adapt to unpredictable wave conditions and perform complex maneuvers [1]. Maintaining stability on constantly shifting wave surfaces, precisely regulating displacement of the center of mass, and making rapid postural adjustments are essential for executing high-difficulty surfing techniques [2, 3]. This activity places considerable demands on multiple facets of physical fitness, with balance ability being one of the most critical components [4]. As noted by Ahmadpoor and Bragazzi, strength training provides a physiological foundation for dynamic balance control. It enhances core and lower limb muscle strength, and optimizes neuromuscular coordination efficiency—such as improving motor neuron excitability and intermuscular coordination. This is especially critical in surfing, where every center-of-mass adjustment and postural correction relies on the synergy between muscular strength and neural regulation [5]. Well-developed balance allows athletes to respond more effectively to wave variations, mitigate the incidence of falls and injuries, and ultimately improve overall performance and competitive outcomes [6]. Consequently, targeted balance training is fundamentally important for surfers across all skill levels.

Despite continuous advancements in balance training methods within sports science, traditional approaches demonstrate substantial limitations in addressing the unique demands of surfing. Most existing protocols emphasize static training on stable surfaces, focusing primarily on foundational stability while failing to simulate the multidirectional instability characteristic of ocean waves [7]. This discrepancy leads to limited transferability to real-world surfing scenarios, where athletes must continuously adapt to sudden, three-dimensional wave perturbations [8]. Furthermore, research dedicated to surfing-specific balance training remains relatively scarce. Although some studies have explored general balance exercises, they often overlook sport-specific motor patterns such as lateral weight transfer during turns or rapid postural recovery following wipeouts [9]. These shortcomings are particularly evident in high-performance settings. A salient example is the Chinese National Surfing Team, whose elite athletes exhibit balance control requirements that exceed the capabilities of conventional standardized programs—designed for average performers rather than competitive surfers facing the intensity and variability of international competitions. Crucially, balance intervention research involving elite surfing athletes is severely limited. The majority of extant literature focuses on recreational surfers or other sports, creating a significant disconnect between available training recommendations and the actual physiological and technical demands of world-class surfing [10]. This gap is especially pronounced concerning the Chinese National Surfing Team, underscoring the urgent need for tailored research to optimize balance training for elite surfing populations [11].

Total Resistance Exercise (TRX) is a form of suspension training that utilizes a controllable unstable environment to enhance proprioception, neuromuscular control, and joint stability [12, 13]. It has been demonstrated to improve balance performance across various sports, such as dynamic balance in track and field athletes [14], in-water stability in swimmers [15], and movement control in soccer players [16]. Nevertheless, the application of TRX in surfing-specific training remains underexplored. Importantly, the system’s design allows it to simulate wave-like perturbations and offers adjustable difficulty levels, making it particularly suitable for addressing the sport-specific balance demands encountered in surfing.

The study implemented TRX suspension training as an intervention to improve balance in elite surfers. All participants were active athletes selected from the Chinese National Surfing Team, representing the highest competitive level in China. The research aimed to compare the effects of TRX suspension training versus conventional training methods on surfers’ balance performance. It was hypothesized that TRX training would lead to greater improvements in balance ability, owing to its capacity to simulate the sport-specific instability encountered during surfing [17, 18]. Furthermore, it was hypothesized that static balance—as a foundational motor capacity—would demonstrate a strong positive correlation with sport-specific balance, whereas dynamic balance (particularly multidirectional control) would exhibit a moderate positive correlation. This expected outcome reflects a hierarchical construct in which fundamental balance capacities synergistically support specialized surfing performance. The findings of this study are anticipated to provide an empirical basis for optimizing balance training protocols in elite surfing athletes and to enhance their preparedness for international competition.

## Methods

### Participants (Initial *n* = 36; 4 withdrew for personal reasons; final *n* = 32)

A priori sample size estimation was conducted using G*Power 3.1.9.2 (Heinrich-Heine-Universität Düsseldorf, Düsseldorf, Germany). As this study aimed to compare the effects of two distinct training modalities on balance performance, the sample size calculation was based on between-group mean differences. With reference to previous balance intervention studies in sports [19], a medium effect size (cohen’s d = 0.5) was assumed, with a significance level a significant level of α = 0.05 and statistical power of 0.80. The analysis indicated that a minimum of 26 participants would be required. As the study participants were elite surfers from the Chinese National Surfing Team, all active team members were invited to participate in screening to ensure representative sampling of the target population and minimize selection bias.

Given the high demands of surfing on lower-limb stability, dynamic balance, and neuromuscular control—particularly under wave-induced perturbations—the inclusion and exclusion criteria were designed to select participants capable of safely completing balance-oriented training while controlling for potential confounders such as prior injury history. The inclusion criteria were as follows: (a) all athletes underwent a pre-experiment physical examination confirming absence of sports-related injuries or illnesses and normal motor function; (b) competitive performance: individual national ranking within the top 12, or team ranking within the top 8 in national-level events (e.g., National Games, National Championships). The exclusion criteria comprised: (a) history of orthopedic surgery (e.g., knee or ankle), fractures, or other musculoskeletal injuries affecting the lower limbs; (b) any lower-limb injury within the preceding three months, or existing conditions that could impair balance; (c) participation in structured balance training interventions within one month prior to the study, to minimize carryover effects. After screening, 36 eligible athletes from the Chinese National Surfing Team were enrolled. During the 8-week intervention period, 4 participants withdrew due to personal reasons, resulting in a final cohort of 32 athletes, including 19 national elite-level and 13 first-class athletes. National elite athletes represent the highest competitive level in Chinese surfing, comparable to international standards, while first-class athletes are also highly skilled and recognized within the national sports system.

To minimize performance and detection bias, this study incorporated blinding measures at both participant and assessor levels. Due to the inherent dissimilarity between TRX suspension training (which requires specialized equipment) and traditional balance training (no specialized equipment), full participant blinding was not feasible. Nevertheless, multiple strategies were implemented to reduce expectation bias and limit exposure to grouping cues, while full blinding was maintained for assessors. The strategy is as follows:

#### Environmental and equipment standardization

 Training was conducted in two separate rooms with identical layout, ambient conditions (temperature: 20–27°, humidity: 68%−78%, low-noise environment), and baseline equipment (e.g., mats, mirrors). For the TRX group, suspension straps were installed by dedicated staff 10 min before each session and removed immediately afterward. No suspension equipment was present at any time in the traditional balance (TB) group training area. Accessories such as resistance bands and timers were stored in uniformly opaque containers to avoid visual identification of group assignment.

#### Procedural standardization

 Participants were assigned randomized daily training time slots to prevent association between session timing and intervention group. All participants wore non-branded, identical training apparel. Warm-up (5-minute dynamic stretching) and cool-down (5-minute static stretching) routines were identical in both groups and conducted by the same strength and conditioning coaches to avoid procedural disparities that could reveal group allocation.

#### Information control

Throughout the recruitment and intervention phases, participants were informed only that the study aimed to “compare two novel balance training protocols designed for elite surfers”. Specific terms such as “TRX”, “suspension training” or “traditional training” were prohibited. Inquiries regarding protocol differences were addressed with a standardized response: “Both protocols are designed to improve balance through distinct mechanisms. Detailed results will be shared after the study.” This approach reduced the risk of unintentional unblinding and minimized expectation bias.

#### Assessor blinding

 Balance assessments were conducted by evaluators who were not involved in training delivery and were blinded to group allocation. Testing took place in a dedicated laboratory separate from training areas. Participants were instructed not to discuss any aspect of their training with the assessors. Only de-identified participant codes were provided during evaluations to ensure complete blinding.

To ensure balance allocation across all baseline characteristics, participants were randomly assigned to either the TRX (TRX suspension training) group or the TB (Traditional Balance training) group using a block randomization procedure, generated via a computerized random number generator in SPSS Statistics (Version 26). Stratification was applied based on age, height, weight, and years of training to enhance baseline comparability between groups. A block size of 4 was selected to accommodate an equal gender distribution (2 males and 2 females per block). After randomization, each group comprised 16 participants (8 males and 8 females). Independent samples t-tests confirmed that there were no significant differences between the TRX and TB groups at baseline in terms of age (15 ± 2 years vs. 15 ± 2 years), height (164.69 ± 7.78 cm vs. 164.69 ± 7.24 cm), weight (52.81 ± 7.64 kg vs. 52.31 ± 8.08 kg), and training years (2.88 ± 0.48 years vs. 2.88 ± 0.7 years) (all *p* > 0.05).

Although the initial sample size calculation indicated a requirement of 26 participants, the final analysis included 32 athletes. A post hoc power analysis confirmed that this sample size provided sufficient statistical power (1-β > 0.80) for detecting meaningful group differences.

Written informed consent was obtained from all adult participants for the publication of personal, clinical, and image data. For minors under 18 years of age, written consent was provided by parents or legal guardians. All participants were fully informed about the study purposes and procedures and voluntarily provided consent. The study protocol was approved by the Ethics Committee of Wuhan Sports University (No. 2021030) and conducted in accordance with the principles of the Declaration of Helsinki. This randomized controlled trial was conducted in accordance with the CONSORT (Consolidated Standards of Reporting Trials) guidelines. The CONSORT checklist is available as supplementary material.

#### Note

 Informal post-study surveys conducted after data collection indicated that none of the participants were able to identify the specific type of training they had received based on content or equipment, supporting the effectiveness of the blinding strategies employed.

### Intervention procedure

The study was conducted during the 2022 training season of the Chinese National Surfing Team. During the 8-week intervention, all athletes followed the Chinese National Surfing Team’s standardized daily schedule to minimize extraneous variability: daytime on-water training, and evening sessions of either the assigned intervention (TRX/TB balance training) or technical video analysis. Throughout the study, training intensity, diet and sleep (consistent timing) were unified between groups. No differences existed except for the balance training interventions, eliminating confounding factors from inconsistent training, diet, or lifestyle. The experiment took place in the Physical Training Room of the Chinese National Surfing Team, located in Lingshui Autonomous County, Hainan Province, China. All training activities—including fitness and surfing practice—were centrally organized and supervised by the national team coaching staff to ensure consistency across participants and control for potential confounding factors. This centralized management ensured uniformity in all daily routines, with the only systematic difference being the balance training intervention assigned to each group. The study employed a “1 + 8” experimental timeline, comprising a one-week adaptation period followed by an eight-week formal intervention. The adaptation phase aimed to familiarize participants with the training protocols and standardize movement execution, thereby ensuring accuracy and consistency during the formal study. Training load was objectively quantified using continuous heart rate (HR) monitoring via Polar H10 sensors. Individual target HR zones were established based on real-time responses to standardized movements, set at 60–80% of age-predicted maximum HR (HR max = 220 – age), consistent with recommended intensity levels for balance training in elite athletes [20]. For each participant, the number of repetitions or duration required to maintain HR within this target zone was determined and used to calibrate individualized training loads for the formal intervention. Daily adjustments were made based on HR data to ensure objective equivalence of training stimulus between groups [21].

The formal intervention period spanned 8 weeks, with training sessions conducted three times per week, each lasting approximately 30 min. Both groups performed balance and core training programs designed in accordance with the principle of progressive overload [22, 23]. The 8-week training protocol was divided into three distinct phases, with intensity progressively increased by elevating target heart rate zones: Phase E1 (Weeks 1–2, Basic Adaptation): Emphasis on static balance exercises, targeting a heart rate zone of 60–70% of HRmax to facilitate neuromuscular adaptation and control [24]. Phase E2 (Weeks 3–5, Quality Enhancement): Introduction of combined static-dynamic exercises, with heart rate maintained at 65–75% of HRmax to elevate metabolic and neuromuscular demands. Phase E3 (Weeks 6–8, Strengthening & Consolidation): Dominated by dynamic balance tasks under conditions simulating competitive stress, with a target heart rate zone of 70–80% of HRmax.

Throughout the intervention, participants were strictly prohibited from engaging in any additional balance-related training outside the prescribed protocol. The detailed training programs for both groups are provided in Table 1. All sessions were supervised by certified strength and conditioning coaches from the national team, with the principal investigator also present to ensure strict adherence to the study protocol.

**Table 1:** Training contents

	TRX Group Training Maneuvers	TB Group Training Maneuvers	Training Parameters
**E1**	Suspended prone planks Suspended legs supine hip thrust Suspended lateral one-handed brace Suspended plank balance	Supine leg lowers Plank Hip thrust Side plank	20–40 s/set (each side) 4 sets 60 s break between sets
**E2**	Suspended prone legs supported open and close, Forward and backward movement of the suspension plate support, Suspended single leg V-support, Suspended prone support group, Suspended straight leg flexion	Plate with translations Knee-to-Chest crunches Straight-Leg Toe-Touch crunches Side crunches Bicycle crunches	**Static maneuvers**: 30–50 s/set **Dynamic maneuvers**: 12–16 reps/set (each side) 4 sets 60 s break between sets
**E3**	Suspended single-legged double-armed support prone leg tucks, Suspended single-legged one-handed lateral support leg suction, Suspended single-legged prone support lateral knee lifts, Suspended prone support knee lift runs, Suspended prone one-handed rotations	Supine double leg and torso raises Single-arm plank with torso rotations Prone single-hand support with knee drives Supine tuck crunches Prone mountain climbers	**Continuous maneuvers**: 40–60 s/set **Dynamic maneuvers**: 16–20 reps/set (each side) 4 sets 60 s break between sets

### Test indicators

All assessments were conducted in a standardized environment within the Physical Training Room of the Chinese National Surfing Team in Lingshui County, Hainan Province. Environmental conditions—including temperature (20–27°), humidity (68%−78%), and noise control—were strictly regulated. Testing sessions were consistently carried out between 7:00 PM to 10:00 PM to minimize diurnal variation. To prevent inter-participant communication and potential bias, athletes were assessed individually, with each subsequent participant entering only after the previous one had fully completed all tests. Two trained assessors were assigned to each outcome measure and remained consistent throughout the study to ensure accuracy and reliability of data collection.

This study evaluated balance ability across three dimensions: static balance, dynamic balance, and surfing-specific balance.

**Fig. 1:**
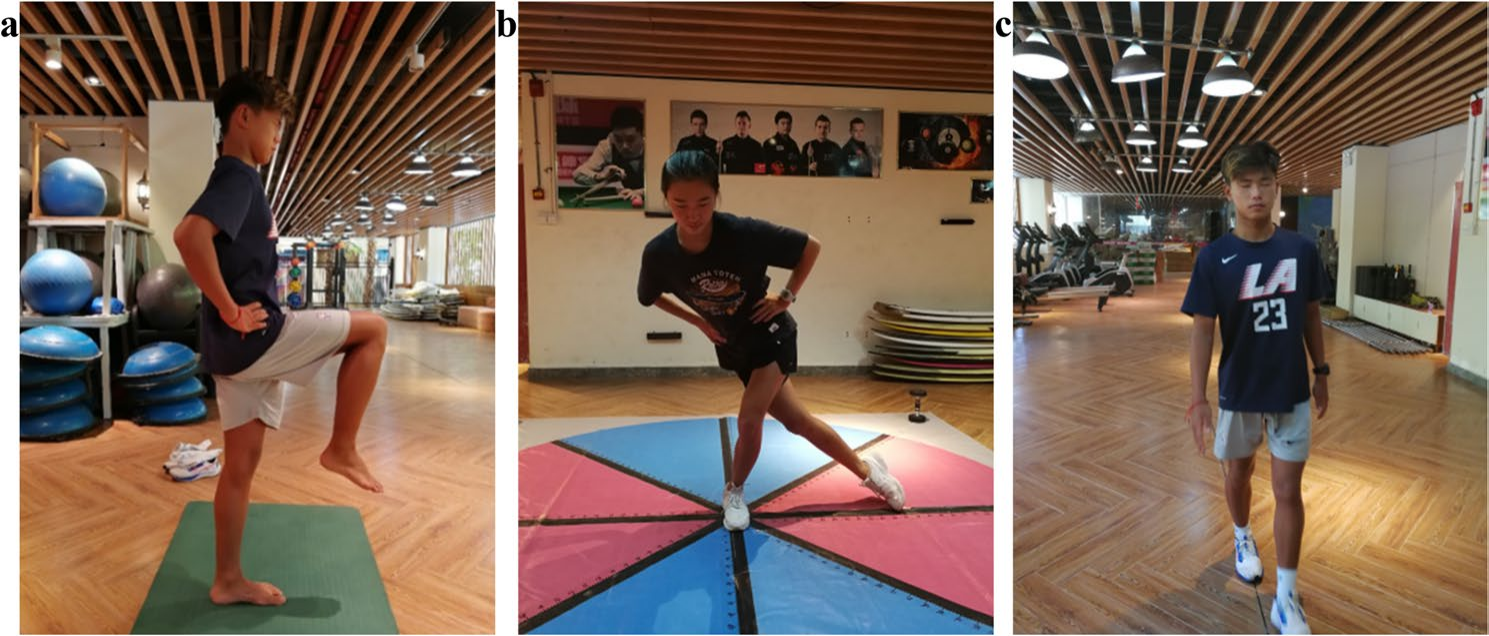
Graphical schematics of the balance assessment protocols (**a**: Eye-closed one-leg stand test, **b**: SEBT, **c**: Linear travel deviation test)

Static balance was assessed using the Eye-closed one-leg stand test [25], a well-validated measure for evaluating static postural stability in athletic populations. For dynamic balance, two established tools were employed: the Star Excursion Balance Test (SEBT), which assesses reach distance in multiple directions under dynamic conditions [26, 27]; and the Linear Travel Deviation Test, which quantifies balance control during linear locomotion: participants walked forward and backward with eyes closed along a 10-meter straight line, with the outcome as average deviation distance (in cm; smaller values indicating better dynamic balance) [28, 29]. Schematic diagrams of the test maneuvers are presented in Fig. 1.

**Fig. 2:**
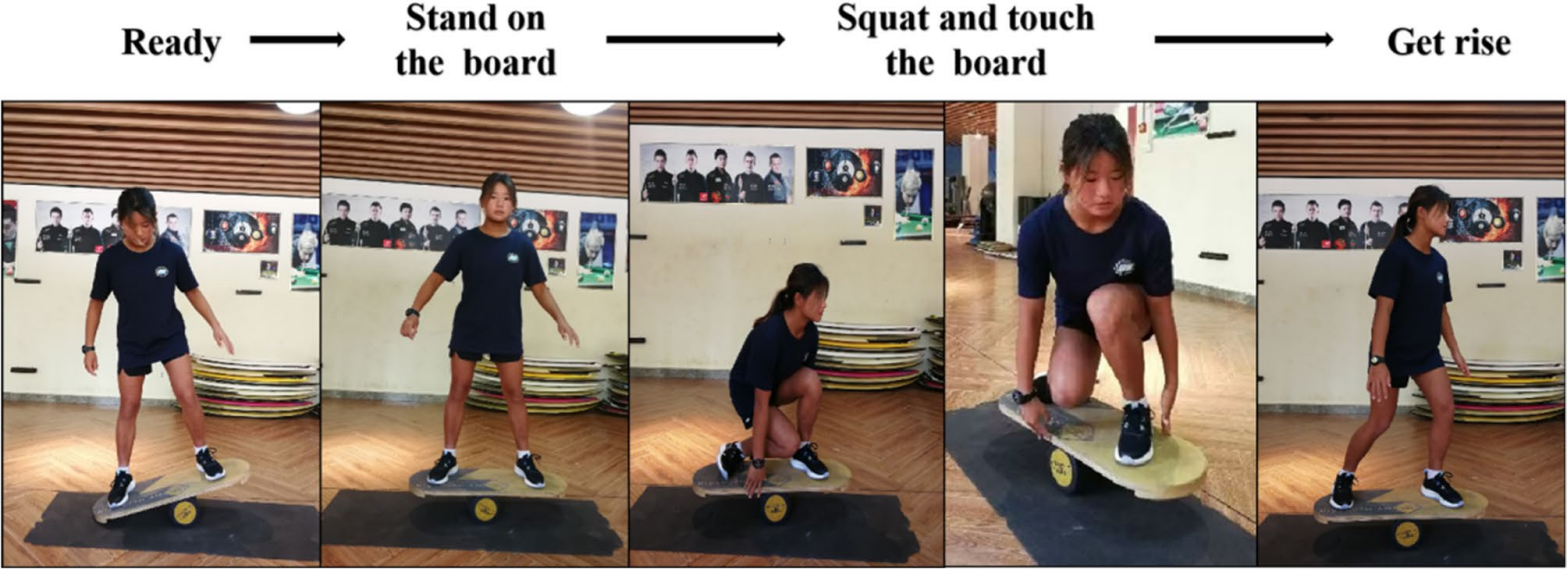
Schematic diagram of the balance board for measuring the direction of the squatting movement

Surfing-specific balance assessment requires particular consideration, as the defining feature of surfing—unpredictable, wave-driven instability—is inherently difficult to reproduce under controlled conditions. Traditional methods for evaluating surfing balance are associated with notable limitations: subjective rating scales, previously common in such studies, are prone to bias due to potential misinterpretation of participant-reported perceptions [30].To address this issue, land-based simulations of aquatic movements have been validated as a reliable alternative for replicating the balance demands specific to surfing [31]. In line with this approach, and building upon the foundational work of Liu et al. (2020) combined with practical insights from the national team’s training and evaluation systems, the Indo board lateral squat was selected as the measure for surfing-specific balance [32]. The indicator emulates fundamental surfing techniques—such as the pop-up, bottom-turn, and cut-back—by integrating lateral squats, deep squats, and rapid rising movements on the Indo board. Performance was quantified as the number of correctly executed lateral squats completed within a fixed time period, with higher scores indicating better balance control, postural adjustment speed, and movement accuracy under unstable conditions. The test maneuver is illustrated in Fig. 2.

### Statistical analyses

Data were were performed using SPSS statistical 26 software (Version 26.0, IBM Corporation, Armonk, New York, USA). Continuous variables are expressed as mean ± standard deviation (X ± SD). The normality of all variables was confirmed by the Shapiro-Wilk test, and homogeneity of variance was verified using Levene’s test both before and after the intervention (all *p* > 0.05), supporting the use of parametric methods. A two-way repeated-measures ANOVA analysis was conducted to examine the main and interaction effects of group (TRX vs. TB) and time (pre- vs. post-intervention) on static, dynamic, and surfing-specific balance performance, with particular emphasis on the Group × Time interaction. Effect sizes were reported as partial eta-squared ($\:{\eta}_{\text{p}}^{\text{2}}$) for ANOVA effects and Cohen’s d for pairwise comparisons, along with 95% confidence intervals (CIs) for mean differences to indicate practical significance. Post-hoc analyses with Bonferroni adjustment were performed for any significant interaction effects. Specifically, paired-samples t-tests were used to analyze within-group differences (i.e., pre- vs. post-intervention comparisons for the TRX group and TB group separately), and independent-samples t-tests were employed to examine between-group differences in post-intervention data; all these post-hoc tests were adjusted via the Bonferroni method to control for Type I errors arising from multiple comparisons. Pearson correlation analysis was used to examine the relationships among changes in static, dynamic, and surfing-specific balance. The significance threshold was set at *p* < 0.05.

## Results

### Effects of 8 weeks of intervention on static balance ability

As shown in Tables 2 and 3, for both left and right leg stances, static balance ability improved significantly post - intervention (main effect of time: left leg F = 48.23, $\:{\eta}_{\text{p}}^{\text{2}}$=0.605; right leg F=52.34, $\:{\eta}_{\text{p}}^{\text{2}}$=0.632; both *p*<0.01, large effect size).

**Table 2:** Influence of an 8 - week intervention on static balance ability measured by eyes - closed single - leg (left foot) stance

Analysis Dimension	Effect Term	Statistic (F/t)	*p*-value	Effect Size ($\:{\eta}_{\text{p}}^{\text{2}}$/Cohen’s d)	95%CI (s)
Two-way Repeated-Measures ANOVA	Main effect of Time	F = 48.23	< 0.001	$\:{\eta}_{\text{p}}^{\text{2}}$=0.605	-
	Group×Time	F = 11.27	< 0.001	$\:{\eta}_{\text{p}}^{\text{2}}$=0.268	-
	Main effect of Group	F = 0.32	0.575	$\:{\eta}_{\text{p}}^{\text{2}}$=0.010	-
Within-Group Comparisons (Pre vs. Post)	TRX group	t=−10.56	< 0.001	Cohen’s d = 1.78	[75.21, 121.79]
	TB group	t=−8.34	< 0.001	Cohen’s d = 1.02	[42.34, 78.16]
Between-Group Comparison (Post-intervention)	TRX vs. TB	t = 3.89	< 0.001	Cohen’s d = 1.15	[20.34, 56.16]

TRX-Pre test: 57.75 ± 38.61, TB-Pre tset: 58.00 ± 32.38. TRX-Post test: 157.38 ± 112.98, TB-Pre tset: 109.81 ± 40.59

**Table 3:** Influence of an 8 - week intervention on static balance ability measured by eyes - closed single - leg (right foot) stance

Analysis Dimension	Effect Term	Statistic (F/t)	*p*-value	Effect Size ($\:{\eta}_{\text{p}}^{\text{2}}$/Cohen’s d)	95%CI (s)
Two-way Repeated-Measures ANOVA	Main effect of Time	F = 52.34	< 0.001	$\:{\eta}_{\text{p}}^{\text{2}}$=0.632	-
	Group×Time	F = 11.87	< 0.001	$\:{\eta}_{\text{p}}^{\text{2}}$=0.286	-
	Main effect of Group	F = 0.34	0.562	$\:{\eta}_{\text{p}}^{\text{2}}$=0.011	-
Within-Group Comparisons (Pre vs. Post)	TRX group	t=−10.23	< 0.001	Cohen’s d = 1.82	[50.21, 80.55]
	TB group	t=−7.89	< 0.001	Cohen’s d = 0.95	[20.34, 43.90]
Between-Group Comparison (Post-intervention)	TRX vs. TB	t = 3.56	< 0.001	Cohen’s d = 1.02	[18.34, 48.18]

TRX-Pre test: 47.50 ± 34.21, TB-Pre tset: 47.94 ± 27.23. TRX-Post test: 158.06 ± 101.68, TB-Pre tset: 107.69 ± 39.38

TRX group outperformed the TB group: Significant Group×Time interactions were observed (left leg F = 11.27, $\:{\eta}_{\text{p}}^{\text{2}}$=0.268; right leg F=11.87, $\:{\eta}_{\text{p}}^{\text{2}}$=0.286; both *p*<0.001, large effect size), with no pre - intervention baseline differences (main effect of group *p*>0.05). Within - group comparisons showed larger improvements in the TRX group (Cohen’s d=1.78 & 1.82) than the TB group (d=0.95 & 1.02). Post - intervention between - group comparisons revealed the TRX group performed significantly better (left leg t=3.89, right leg t=3.56, both *p*<0.001), with differences remaining significant after Bonferroni correction (*p*<0.001).

The intervention led to a significant improvement in surfers’ static balance ability. Furthermore, the TRX group demonstrated superior improvement compared to the TB group in both left and right limb stability.

### Effects of 8 weeks of intervention on dynamic balance ability

The findings are presented in Tables 4 and 5, and 6. Prior to the intervention, no significant differences were observed between the two groups in the SEBT left-leg stance, SEBT right-leg stance (both *p* > 0.5), or Linear travel deviation tests (*p* = 0.525). After Bonferroni correction, these non - significant results still hold, which further validates this baseline equivalence. This baseline equivalence ensures that subsequent disparities in outcomes can be attributed to the inherent differences between the training programs.

**Table 4:** Influence of an 8 - week intervention on dynamic balance ability measured by SEBT (left foot support)

Analysis Dimension	Effect Term	Statistic (F/t)	*p*-value	Effect Size ($\:{\eta}_{\text{p}}^{\text{2}}$/Cohen’s d)	95%CI (%)
Two-way Repeated-Measures ANOVA	Main effect of Time	F = 41.37	< 0.001	$\:{\eta}_{\text{p}}^{\text{2}}$=0.582	-
	Group×Time	F = 8.72	0.006	$\:{\eta}_{\text{p}}^{\text{2}}$=0.226	-
	Main effect of Group	F = 0.29	0.592	$\:{\eta}_{\text{p}}^{\text{2}}$=0.010	-
Within-Group Comparisons (Pre vs. Post)	TRX group	t=−7.85	< 0.001	Cohen’s d = 1.62	[0.03, 0.07]
	TB group	t=−5.64	< 0.001	Cohen’s d = 1.17	[0.02, 0.05]
Between-Group Comparison (Post-intervention)	TRX vs. TB	t = 2.98	0.005	Cohen’s d = 0.85	[0.01, 0.06]

TRX-Pre test: 1.00 ± 0.05, TB-Pre tset: 0.99 ± 0.05. TRX-Post test: 1.15 ± 0.05, TB-Pre tset: 1.12 ± 0.04

**Table 5:** Influence of an 8 - week intervention on dynamic balance ability measured by SEBT (right foot support)

Analysis Dimension	Effect Term	Statistic (F/t)	*p*-value	Effect Size ($\:{\eta}_{\text{p}}^{\text{2}}$/Cohen’s d)	95%CI (%)
Two-way Repeated-Measures ANOVA	Main effect of Time	F = 38.72	< 0.001	$\:{\eta}_{\text{p}}^{\text{2}}$=0.554	-
	Group×Time	F = 7.64	0.009	$\:{\eta}_{\text{p}}^{\text{2}}$=0.203	-
	Main effect of Group	F = 0.31	0.582	$\:{\eta}_{\text{p}}^{\text{2}}$=0.011	-
Within-Group Comparisons (Pre vs. Post)	TRX group	t=−7.23	< 0.001	Cohen’s d = 1.51	[0.07, 0.13]
	TB group	t=−5.12	< 0.001	Cohen’s d = 1.07	[0.04, 0.09]
Between-Group Comparison (Post-intervention)	TRX vs. TB	t = 2.85	0.007	Cohen’s d = 0.81	[0.02, 0.08]

TRX-Pre test: 0.99 ± 0.07, TB-Pre tset: 0.98 ± 0.05. TRX-Post test: 1.15 ± 0.05, TB-Pre tset: 1.12 ± 0.04

**Table 6:** Influence of an 8 - week intervention on dynamic balance ability measured by linear travel deviation test

Analysis Dimension	Effect Term	Statistic (F/t)	*p*-value	Effect Size ($\:{\eta}_{\text{p}}^{\text{2}}$/Cohen’s d)	95%CI (cm)
Two-way Repeated-Measures ANOVA	Main effect of Time	F = 57.29	< 0.001	$\:{\eta}_{\text{p}}^{\text{2}}$=0.654	-
	Group×Time	F = 12.47	< 0.001	$\:{\eta}_{\text{p}}^{\text{2}}$=0.302	-
	Main effect of Group	F = 0.41	0.525	$\:{\eta}_{\text{p}}^{\text{2}}$=0.014	-
Within-Group Comparisons (Pre vs. Post)	TRX group	t = 8.36	< 0.001	Cohen’s d = 1.91	[61.34, 78.66]
	TB group	t = 6.72	< 0.001	Cohen’s d = 1.40	[38.52, 55.48]
Between-Group Comparison (Post-intervention)	TRX vs. TB	t=−3.87	< 0.001	Cohen’s d = 1.10	[−12.38, −3.62]

(1)TRX-Pre test: 69.27 ± 23.52, TB-Pre tset: 68.67 ± 24.73. TRX-Post test: 12.03 ± 6.27, TB-Pre tset: 20.08 ± 5.12. (2) A reduction in deviation score indicates improvement

Efficacy of training interventions: Post-intervention, significant improvements in dynamic balance ability were evident across all assessments (main effect of time, *p* < 0.001). After Bonferroni correction, the significance of these improvements in dynamic balance ability remains (*p* < 0.001). Large effect sizes were observed for SEBT left-leg stance ($\:{\eta}_{\text{p}}^{\text{2}}$=0.582), SEBT right-leg stance ($\:{\eta}_{\text{p}}^{\text{2}}$=0.554), and Linear travel deviation ($\:{\eta}_{\text{p}}^{\text{2}}$=0.654), confirming that both training protocols effectively enhanced dynamic balance control.

Superiority of the TRX program: A significant Group×Time interaction was detected (*p* < 0.01). After Bonferroni correction, this significant interaction still exists (*p* < 0.01), with the TRX group demonstrating greater improvements. Specifically, the TRX group achieved significantly higher post-intervention scores than the TB group in the SEBT left-leg stance (*p* = 0.005, d = 0.85) and SEBT right-leg stance (*p* = 0.007, d = 0.81), and a more pronounced reduction in Linear travel deviation (*p* < 0.001, d = 1.10). After Bonferroni correction, these significant differences between the two groups in the post - intervention results still remain significant.

In summary, the TRX suspension training group demonstrated significantly greater improvements in dynamic balance ability compared to the TB group, as evidenced by superior performance in both the SEBT and the Linear travel deviation control.

### Effects of 8 weeks of intervention on surfing-specific balance ability

Prior to the intervention, there was no significant difference in the number of Indo board side squats completed per unit time between the two groups (main effect of group, F = 0.38, *p* = 0.540). After Bonferroni correction, this non - significant result still holds (*p* = 0.540). As shown in the Table 7, this result eliminates the interference of baseline levels on subsequent intervention effects, ensuring that differences in training programs are the core inducement for outcome divergence and laying a foundation for the reliability of the research conclusions.

**Table 7:** Influence of an 8 - week intervention on dynamic balance ability measured by Indo board lateral squat

Analysis Dimension	Effect Term	Statistic (F/t)	*p*-value	Effect Size ($\:{\eta}_{\text{p}}^{\text{2}}$/Cohen’s d)	95%CI (times)
Two-way Repeated-Measures ANOVA	Main effect of Time	F = 68.42	< 0.001	$\:{\eta}_{\text{p}}^{\text{2}}$=0.701	-
	Group×Time	F = 10.35	< 0.001	$\:{\eta}_{\text{p}}^{\text{2}}$=0.258	-
	Main effect of Group	F = 0.38	0.540	$\:{\eta}_{\text{p}}^{\text{2}}$=0.013	-
Within-Group Comparisons (Pre vs. Post)	TRX group	t=−9.21	< 0.001	Cohen’s d = 2.11	[18.32, 25.68]
	TB group	t=−7.14	< 0.001	Cohen’s d = 1.52	[12.45, 19.55]
Between-Group Comparison (Post-intervention)	TRX vs. TB	t = 3.69	< 0.001	Cohen’s d = 1.06	[3.28, 10.72]

TRX-Pre test: 29.06 ± 4.19, TB-Pre tset: 29.38 ± 4.32. TRX-Post test: 50.13 ± 5.32, TB-Pre tset: 43.69 ± 3.61

Validation of Training Efficacy: After the intervention, the number of Indo board side squats completed per unit time significantly increased in both groups (main effect of time, F = 68.42, *p* < 0.001, $\:{\eta}_{\text{p}}^{\text{2}}$=0.701). After Bonferroni correction, the significance of this increase remains (*p*<0.001). The large effect size fully confirms that the training can effectively enhance the sport - specific balance ability of surfers, providing a basic basis for the practical value of the training programs.

Superiority of the TRX Program: A significant Group×Time interaction was detected (F = 10.35, *p* < 0.001, $\:{\eta}_{\text{p}}^{\text{2}}$=0.258), confirming distinct differences in training efficacy between the TRX and TB groups; this significance persisted following Bonferroni correction (*p*<0.001). Within-group analyses revealed that the TRX group exhibited a large magnitude of effect for improvements in completion counts (t=−9.21, d=2.11), and the TB group both showed the large effect (t=−7.14, d=1.52); both effects remained statistically significant post-Bonferroni correction (*p*<0.001 for both). Further post-intervention between-group comparisons verified that the TRX group completed significantly more lateral squats than the TB group (t=3.69, *p*<0.001, d=1.06), with this significant difference also retained after Bonferroni correction. Collectively, these findings demonstrate that TRX training confers greater advantages in enhancing surfers’ sport-specific balance, as measured by the Indo board lateral squat test.

### Correlation analysis of static and dynamic balance ability with surfing-specific balance ability

Surfing-specific balance, a critical determinant of surfing performance, is influenced by both static and dynamic balance capacities. To quantify their relative contributions to sport-specific performance, this study employed Pearson correlation analysis to examine the relationships between improvements in each balance dimension and gains in surfing-specific balance. The objective was to identify which balance component had the strongest association with sport-specific performance, thereby providing evidence for more targeted training interventions.

**Fig. 3:**
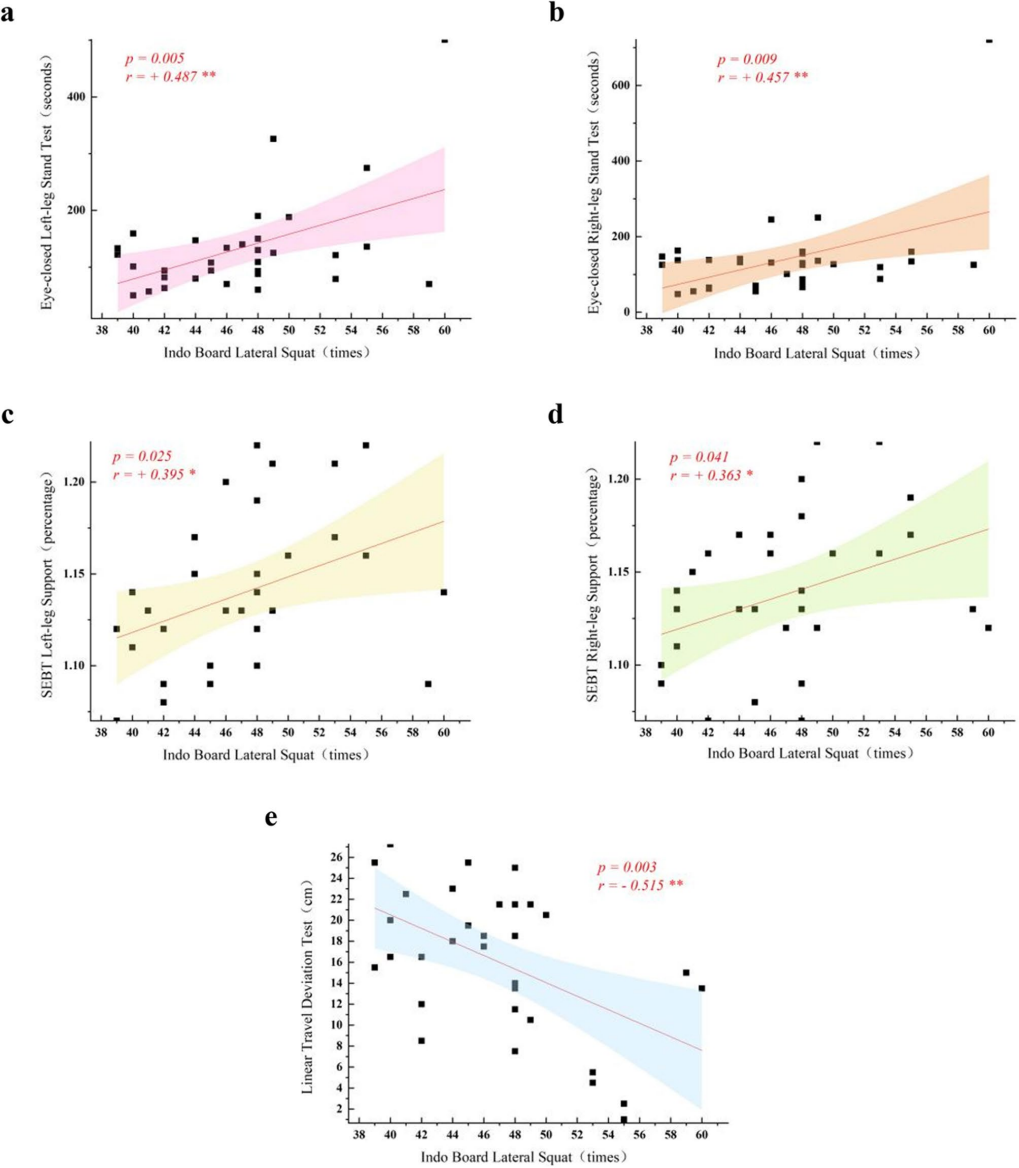
Correlations between static and dynamic balance skills with specialized balance skills (**a**: eye-closed left-footed stance, **b**: eye-closed right-footed stance, **c**: SEBT left-legged bracing, **d**: SEBT right-legged bracing, and **e**: Linear travel deviation)

Pearson correlation analysis revealed that changes in surfing-specific balance were significantly correlated with changes in both static and dynamic balance (*p* < 0.05). Specifically:

Changes in surfing-specific balance exhibited a highly significant moderate positive correlation with changes in static balance (*p*_*left*_ =0.005, *r*_*left*_ = + 0.487, 95% CI [0.213, 0.692]; *p*_*right*_ =0.009, *r*_*right*_ = + 0.457, 95% CI [0.178, 0.675]; Fig. 3a and b). Clinically, this correlation indicates that for every 1 standard deviation (SD) improvement in static balance, surfing-specific balance increases by approximately 0.46–0.49 SD. In practical surfing scenarios, this translates to enhanced stability for athletes while waiting on waves, providing a more solid foundation for critical movements such as pop-ups and turns.

Changes in surfing-specific balance showed a significant weak positive correlation with changes in multidirectional balance and body control within dynamic balance (*p*_*left*_ =0.025, *r*_*left*_ = + 0.395, 95% CI [0.098, 0.631]; *p*_*right*_ =0.041, *r*_*right*_ = + 0.363, 95% CI [0.052, 0.610]; Fig. 3c and d). From a practical perspective, this weak correlation suggests that while the magnitude of surfing-specific balance improvement is limited (approximately 0.36–0.40 SD per 1 SD improvement in multidirectional balance), it still helps athletes adjust their posture more flexibly in irregular waves and reduce the risk of imbalance.

Changes in surfing-specific balance demonstrated a highly significant moderate negative correlation with changes in directional perception and control within dynamic balance (*p* = 0.003, *r* = − 0.515, 95% CI [−0.718, −0.264]; Fig. 3e). In terms of practical value, this negative correlation means that for every 1 SD improvement in directional control accuracy (i.e., reduced directional deviation), surfing-specific balance increases by approximately 0.52 SD. This specifically manifests as athletes reducing energy waste from unnecessary movements in complex wave conditions, enabling more efficient balance maintenance and technical execution.

## Discussion

In surfing, athletes perform diverse technical maneuvers in complex, variable wave conditions, with balance capacity being a core determinant of performance and competitive outcomes. This study compared the effects of TRX suspension training and traditional balance training on static, dynamic, and surfing-specific balance in elite surfers, while investigating relationships between these balance dimensions.

### Analysis of static balance results

Static balance, which serves as a foundational motor capacity in surfing, is critically involved in scenarios such as athletes awaiting waves or maintaining momentary stability on the wave face. In this study, static balance was evaluated using the eyes-closed single-leg stance test. The results indicated that both the TRX and TB groups showed significant improvements following the intervention (*p* < 0.001). However, the TRX group exhibited superior gains ($\:{\eta}_{\text{p}}^{\text{2}}$= 0.268 & 0.286, all *p* < 0.01) with larger effect sizes compared to the TB group. These outcomes align with the initial research hypothesis.

The observed intergroup differences may be mechanistically explained by the divergent neuromuscular and sensorimotor demands imposed by the two training modalities. TRX suspension training introduces an unstable support surface that challenges the body to continuously execute reactive adjustments, thereby facilitating dynamic stabilization through repeated cycles of imbalance and recovery [15, 33]. This process fully activates the vestibular system (a core organ for perceiving spatial position and movement status) and proprioceptors (sensing joint position and muscle tension) [14], thereby optimizing the central nervous system’s efficiency in integrating sensory signals and enhancing the precision of neuromuscular control [34]. Studies have shown that long-term training in such unstable environments can induce changes in neural synaptic plasticity, optimize neural conduction pathways, thereby improving the efficiency of the central nervous system in integrating sensory signals and enhancing the precision of neuromuscular control [35]. In contrast, TB focuses on continuously stimulating muscles around the abdomen, lower back, and lower limb joints through movements such as single-arm support rotation and bicycle crunches, aiming to enhance local muscle strength and provide a stable support base [36]. However, the stable environment of TB training makes it difficult to provide deep stimulation to the vestibular-proprioceptive system, fails to fully activate their coordinated working mechanism, and thus leads to insufficient activation of the higher neural centers responsible for balance regulation.

In the eyes-closed single-leg stance test, the TRX group’s superior performance under the absence of visual feedback not only indicates a stronger capacity to rely on vestibular-proprioceptive cues to suppress body sway—an ability directly relevant to surfing scenarios (e.g., brief stationary moments while waiting for waves or adjusting posture on an unstable surfboard)—but also serves as a direct reflection of the aforementioned mechanism, by demonstrating the central nervous system’s capability to flexibly adjust sensory weighting amid changes in complex sensory input, which is crucial for sustaining balance stability.

In conclusion, TRX suspension training demonstrates superior efficacy over traditional balance training in enhancing the foundational static stability essential for surfing, owing to its pronounced facilitation of vestibular-proprioceptive and neuromuscular integration. These advantages are not only supported by empirical results but are also highly consistent with the sport-specific requirement for maintaining static control within inherently unstable aquatic environments.

### Analysis of dynamic balance results

Dynamic balance, which is essential for surfers to resist multidirectional wave perturbations, maintain linear motion trajectories, and perform technical maneuvers, was assessed in this study using the Linear travel deviation test and the SEBT. The results demonstrated that the TRX group showed significantly greater improvement in dynamic balance after the intervention compared to the TB group (between-group *p* < 0.001, large effect size), which aligns with the research hypothesis.

The advantage of TRX suspension training lies in its high fidelity to the real-world scenario of “unstable support + multidirectional adjustment” during surfing. Throughout the training, athletes are required to frequently switch between imbalance and rebalance, which prompts continuous activation of core stabilizing muscles (e.g., transversus abdominis, pelvic floor muscles) and fully engages the proprioceptive system to achieve precise control of body posture [37, 38]. This neuromuscular control pattern shaped by TRX suspension training is highly congruent with the actual demands of countering wave surges in surfing. This neuromuscular control pattern aligns closely with the principles of functional training proposed by Sheppard, which emphasize that simulating sport-specific unstable environments enhances the transfer of training effects to real-world athletic performance [39]. Specifically, Sheppard’s research team, through systematic analyses across multiple sports disciplines (e.g., team sports, combat sports, and aquatic sports), has confirmed that training programs incorporating multidimensional perturbation adaptation—defined as structured exposure to variable, task-relevant disturbances—are more effective in improving dynamic balance capacity than conventional stable-surface training. This finding provides critical theoretical support for the superior performance of the TRX group observed in the present study, as TRX suspension training inherently integrates multidimensional perturbations (e.g., horizontal and vertical displacements of the support base) that mirror the unstable conditions of surfing.

In contrast, traditional balance training (TB) is primarily performed on fixed and stable surfaces, with an emphasis on isolated muscle strengthening. This approach offers limited stimulus for intermuscular coordination and balance regulation under dynamically unstable conditions [40]. For example, muscular activation during TB tends to be localized to specific regions, failing to elicit comprehensive multidirectional force production and whole-body coordination. As a result, the transferability of TB-acquired adaptations to the complex and unpredictable balance demands of surfing is constrained. Previous studies have indicated that training in unstable environments more effectively improves balance control and promotes muscular synergy during dynamic tasks [41]. The present study further supports this view, as the statistically significant improvements in both the Linear Travel Deviation Test and Star Excursion Balance Test (SEBT) following TRX training provide empirical evidence for the benefits of sport-specific unstable training.

### Analysis of surfing-specific balance results

The Indo board lateral squat test closely mimics sport-specific movement patterns in surfing, such as maintaining board stance and executing directional changes, thereby providing a valid measure of surfing-related balance performance. Results showed that the TRX group completed significantly more Indo board lateral squats per unit time than the TB group (between-group *p* < 0.001), fully confirming the superior effectiveness of TRX suspension training in enhancing surfing-specific balance.

TRX suspension training induces an unstable environment that necessitates continuous adaptation to support conditions analogous to those of a surfboard. This modality effectively strengthens the “rapid adjustment-force production” synergy required in such specialized scenarios [42]. This finding aligns closely with the biomechanical research findings on elite surfers conducted by Furness and colleagues. Specifically, Furness et al., through analyses utilizing motion capture technology, observed that elite surfers maintain center-of-mass stability by means of sustained activation of core stabilizer muscles and precise lower limb force production in wave environments—adaptation processes that TRX training specifically targets for enhancement [43]. Notably, their research further indicates that the high injury rates at certain joints (e.g., shoulder and ankle joints) are directly associated with insufficient sport-specific balance capacity, which provides valuable empirical support for the necessity of emphasizing sport-specific balance training in the present study.

During the Indo board assessment, athletes depend on sustained activation of core stabilizing musculature to preserve center-of-mass stability, while lower extremity muscles generate precise force under instability to execute lateral squats—a mechanism highly consistent with the balance control demands of surfing, where frequent postural adjustments are requisite to counteract wave surges [33]. In contrast, TB, which lacks simulation of unstable environments, exhibits limited transfer of training effects to surfing-specific contexts, resulting in comparatively modest improvements in sport-specific balance. Prior research has established that training modalities replicating real sport scenarios significantly strengthen the association between training outcomes and actual performance [17, 30],—a notion consistent with current sport-specific training paradigms. As emphasized in Dhahbi (2025), sport-specific approaches (e.g., TRX training mimicking the unstable surfboard surface) outperform traditional generalized methods by aligning training stimuli with real sport demands [44]. This further explains the TRX group’s superior performance in the Indo board test, as its surf-mimetic adaptations directly transfer to the test’s surfing-specific balance challenges.

### Theoretical implications of correlation analysis

Pearson correlation analysis was conducted to explore associations between static/dynamic balance and surfing-specific balance. Results revealed that static balance was significantly correlated with surfing-specific balance in a moderate positive manner (*r*_*left*_ = + 0.487, *r*_*right*_ = + 0.457). Dynamic balance subdomains showed distinct relationships: multidirectional balance and body control were significantly correlated with surfing-specific balance in a weak positive manner (*r*_*left*_ = + 0.395, *r*_*right*_ = + 0.363), while directional perception and control exhibited a significant moderate negative correlation with surfing-specific balance (*r* = − 0.515).

These correlations provide empirical support for constructing a “hierarchical model of surfing-specific balance.” Static balance, as the foundational layer, ensures basic postural stability and center-of-mass control during surfing, serving as a prerequisite for advanced sport-specific balance. Multidirectional balance and body control, as the intermediate layer, facilitate adaptive postural adjustments amid multidirectional wave impacts and environmental fluctuations, enabling the execution of technical maneuvers and enhancing surfing-specific performance.

The negative correlation between directional perception/control and surfing-specific balance profoundly reveals the inherent contradiction between the environmental uniqueness of this sport and its control requirements. Traditional directional control relies on a “linear regulation based on preset targets” model, whose effectiveness is built on the consistent input of sensory signals (e.g., visual and proprioceptive cues) in stable environments [45]—a model that is highly effective in land-based sports involving fixed-direction running or jumping. However, in surfing scenarios, wave dynamics exhibit inherent nonlinearity and unpredictability—Farley’s research, via motion capture technology, found that the actual time surfers spend riding waves only accounts for 8% of the total duration, and they must cope with instantaneous changes in wave speed, direction, and force [46]. Such an extremely dynamic environment renders linear control strategies completely ineffective. When athletes apply rigid linear strategies to surfing, a “strategy-environment mismatch” occurs: fixed directional adjustments fail to adapt to real-time wave perturbations, leading to increased body sway and reduced balance stability—which constitutes the core mechanism underlying the negative correlation between the two [47]. As Sheppard noted in his research on surfing balance, the continuous changes in wave environments require the sensorimotor system to possess far greater dynamic adaptability than that needed in land-based sports, and the rigidified patterns of traditional directional control are fundamentally incompatible with this requirement.

In fact, human balance control is inherently a dynamic matching process encompassing “sensory input-central integration-motor output“ [48]. Traditional directional control ability originates from the long-term solidification of “sensory signal pattern-motor response” in stable environments (e.g., the fixed association between directional perception and muscle activation during linear land-based movements); whereas in surfing, non-periodic perturbations induced by waves—such as passive body rotation and center-of-mass shifts caused by surges—disrupt this solidified association. Consequently, athletes must abandon “preset directional control” and switch to an adaptive model characterized by “real-time sensory feedback (vestibular-proprioceptive)-rapid central reorganization-flexible motor adjustment“ [49]. In this context, the stronger an athlete’s traditional directional control ability (i.e., the more rigid their linear regulation pattern), the weaker the adaptive flexibility of their sensorimotor system to “non-periodic perturbations”—and thus, the more pronounced the negative correlation with surfing-specific balance.

Pearson correlation analysis revealed that static balance exhibited a moderate positive correlation with surfing-specific balance, multidirectional balance/body control showed a weak positive correlation, and directional perception/control demonstrated a moderate negative correlation—providing support for the “hierarchical model of surfing-specific balance.”

### Practical value of the hierarchical model: guidance for periodized training of elite surfers

The “hierarchical model of surfing-specific balance” holds both theoretical significance and practical guiding value, enabling the provision of precise prescriptions for the periodized training of elite surfers. Its implementation is specifically realized through three core phases:

First, during the general preparation phase (8–12 weeks pre-competition), the focus should be on strengthening the “foundational layer” of the model—static balance ability. Training methods such as eyes-closed single-leg stance and static support on foam rollers are adopted to improve core stability and the precision of center-of-mass control, laying a solid foundation for subsequent sport-specific training. This design directly aligns with the moderate positive correlation between static balance and surfing-specific balance.

Second, in the specific development phase (4–8 weeks pre-competition), the training shifts to the “intermediate layer” of the model—multidirectional balance and body control ability. Unstable surfaces such as balance pads and Swiss balls are introduced, with exercises including multidirectional squats and dynamic center-of-mass transfer. These practices simulate the multidirectional impact scenarios of waves to enhance the ability of adaptive postural adjustment. This not only responds to the weak positive correlation between this ability and surfing-specific balance but also bridges the gap between basic balance capacity and sport-specific performance.

Finally, in the pre-competition tapering phase (1–4 weeks pre-competition), targeted efforts are made to address the core contradiction in the model—the negative correlation between directional perception/control and surfing-specific balance. Training protocols such as TRX suspension dynamic direction changes and wave pool-simulated random turns are designed to actively disrupt linear directional perception, forcing athletes to adjust movements based on real-time vestibular-proprioceptive feedback and strengthen “non-preset balance control ability,” thereby adapting to the unpredictable wave conditions in competitions.

The core value of TRX suspension training lies in its complete alignment with the training logic of the hierarchical model: the complex unstable environment it simulates not only facilitates the enhancement of multidirectional balance during the specific development phase but also breaks the rigidification of traditional directional control during the pre-competition tapering phase. This explains why the TRX group demonstrated superior performance in the surfing-specific balance test.

### Limitations and future perspectives

Despite the valuable insights provided by this study regarding the efficacy of TRX suspension training in improving surfing-specific balance, several limitations should be acknowledged. First, although a priori and post hoc power analyses supported statistical validity, the relatively small sample size (*n* = 16 per group) may constrain the generalizability of the findings to the broader elite surfer population. Second, despite balanced sex distribution, no subgroup analyses were conducted to examine potential sex-based differences in balance adaptation—a notable omission given established evidence of sex-specific neuromuscular responses. Third, constrained by the fixed training camp schedule of the Chinese National Surfing Team, the intervention period of this study was only set to 8 weeks, and it was not possible to extend the duration for longer-term intervention observations. Additionally, the absence of long-term follow-up data further limits the in-depth analysis of the sustainability of the observed improvements. Fourth, while the Indo board lateral squat test is ecologically relevant to surfing movements, its psychometric properties (e.g., test–retest reliability, normative data) have not been formally validated in elite surfers, which may affect the interpretability of results. Fifth, the study focused solely on balance performance and did not include direct measures of competitive surfing performance (e.g., maneuver scores, board control metrics), thereby limiting conclusions regarding translational benefits. Finally, the lack of neuromuscular monitoring (e.g., surface EMG, motion capture) precluded mechanistic analysis of how balance enhancement occurred at the physiological level.

To address these limitations, future studies should consider: expanding the sample size and incorporating sex-specific subgroup analyses; extending the intervention duration and implementing longer-term follow-up assessments; validating the Indo board test in elite surfing populations; integrating sport-specific performance outcomes; and employing advanced biomechanical and physiological measurements to elucidate underlying mechanisms. These steps would help establish more robust, evidence-based training recommendations for high-performance surfers.

## Conclusions

This study demonstrates that TRX suspension training represents a highly effective method for improving static, dynamic, and surfing-specific balance in competitive surfers. These findings offer both empirical support and a theoretical foundation for evidence-based design of sport-specific balance training regimens. Furthermore, the results indicate that static balance acts as a foundational ability, while dynamic balance serves as an advanced motor competency—together forming an integral basis for surfing-specific balance performance. The outcomes also provide strong empirical validation for the application of the principle of specificity in surfing training, supporting a shift in training methodology from general balance conditioning toward scenario-based, sport-specific simulation approaches.

In practical terms, TRX suspension training can be integrated into elite surfers’ pre-competition specific preparation or off-season balance enhancement sessions: it reduces the risk of injury from imbalance in waves by strengthening core and lower limb stability, while also improving the stability of technical maneuvers in complex wave conditions to further optimize competitive performance.

## Supplementary Information

Supplementary Material 1.

## Data Availability

The raw data supporting the conclusion of this article will be made available by the authors, without undue reservation.
