# Supplementary figures and images for: Effects of TRX suspension training versus traditional balance training on balance performance in elite surfers
Source: BMC Sports Sci Med Rehabil. 2025 Nov 24;17:352. doi: 10.1186/s13102-025-01411-z (PMC12642247; doi:10.1186/s13102-025-01411-z)

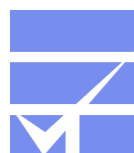

## CONSORT 2010 Flow Diagram

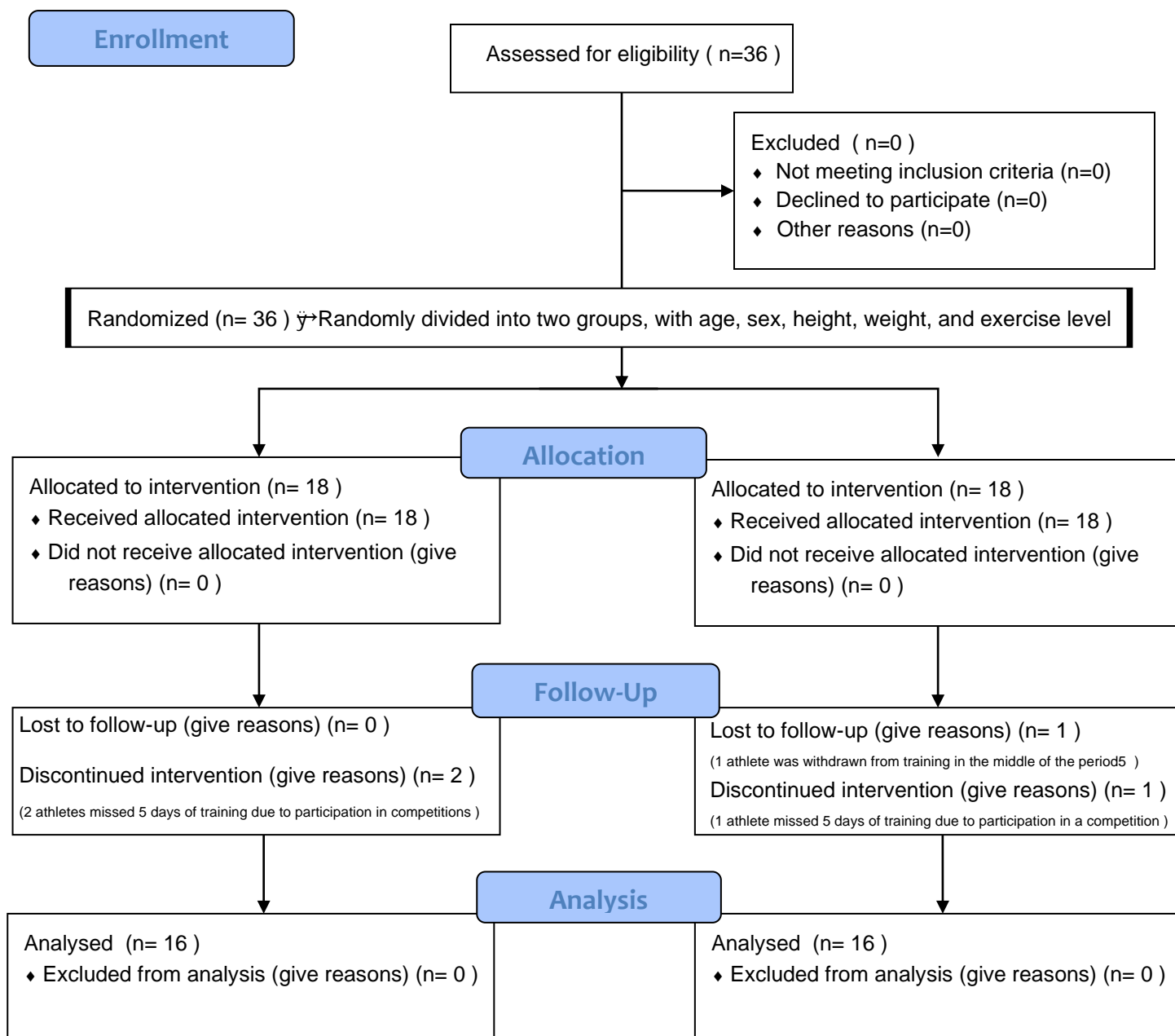

Supplement: Supplementary file 1 — Supplementary Material 1. [file 13102_2025_1411_MOESM1_ESM.pdf]
